# Risk Factors for Predicting Mortality and Amputation of Patients with Necrotizing Soft-Tissue Infections: Retrospective Analysis of 111 Cases from a Single Medical Center

**DOI:** 10.1155/2023/6316896

**Published:** 2023-11-11

**Authors:** Hanghui Cen, Ronghua Jin, Jun Yin, Xingang Wang

**Affiliations:** Department of Burns and Wound Repair Surgery, The Second Affiliated Hospital, School of Medicine, Zhejiang University, China

## Abstract

**Objective:**

Necrotizing soft-tissue infections (NSTIs) are rare clinical infections with surgical emergencies having a high mortality rate. This study aimed to investigate risk factors for mortality and amputation of patients with NSTI.

**Methods:**

We retrospectively analyzed critical factors for outcomes of 111 patients with NSTI hospitalized in our department from 1 January 1999 to 31 December 2018. NSTI diagnosis was based on the patient's clinical characteristics, laboratory risk indicator for necrotizing fasciitis (LRINEC) score, laboratory test data, and microbiological findings in blood and wound culture. The risk factors for mortality and amputation of NSTI were determined using univariate or multivariate logistic regression analysis, receiver operating characteristics (ROC), and the area under the ROC curve (AUC) at 90 days after admission.

**Results:**

Diagnosis of 111 patients with NSTI was confirmed according to clinical features, LRINEC score, image data, laboratory findings, and microorganism culture in blood and wounds. The mortality rate was 9.91% (11/111) at day 90 follow-up. High white blood cell (WBC), low hematocrit (HCT), and multiple surgeries were identified to be critical risk factors for NSTI mortality in univariate and multivariate logistic analyses. AUCs, 95% confidence intervals (CI), and *P* values of risk factors were 0.699, 0.54–0.95, and *P* = 0.0117 for high WBC; 0.788, 0.63–0.97, and *P* = 0.0006 for low HCT; and 0.745, 0.59–0.90, and *P* = 0.0018 for multiple surgeries, respectively. These patients also had high LRINEC scores. Amputation occurred in 34.23% (38/111) of patients. Risk factors for amputation were higher age, low hemoglobin (Hb), and multiple wounds. AUCs, 95% confidence intervals (CI), and *P* values were 0.713, 0.11–0.32, and *P* < 0.0001 for higher age; 0.798, 0.08–0.29, and *P*=0.0007 for low Hb; and 0.757, 0.17–0.34, and *P*  < 0.0001 for multiple lesion sites, respectively.

**Conclusions:**

High LRINEC scores, high WBC, low HCT, and multiple surgeries were relevant to increased mortality. Higher age, low Hb, and multiple wounds were associated with amputation risk. These clinical features must be paid attention to when patients are diagnosed with NSTI.

## 1. Introduction

Necrotizing soft-tissue infections (NSTIs) are a group of rare and severe infectious diseases with a high mortality rate [1–3]. The typical features of NSTI include extensive soft tissues necrotizing, including necrotizing fasciitis (NF), myositis, gas gangrene, Fournier gangrene (FG), and systemic signs of toxicity from sepsis to infectious shock [4, 5]. Multiple organs in NSTI are frequently damaged with varying degrees [6–8]. NSTI can be divided into type I NSTI (polymicrobial) and type II NSTI (monomicrobial) according to microbial infection types [1]. The estimated NSTI incidence varies between 0.3 and 15.5/100000 inhabitants from Europe or the USA to Asia [5, 7–11]. In addition, NSTIs have up to 20–35% mortality rate [9, 12–14]. Most patients with NSTI must be admitted into the intensive care unit (ICU) for treatments. The therapeutic methods for NSTI include urgent surgical debridement of infected tissues, broad-spectrum antibiotics use, and protection of organ functions as soon as possible [1, 7]. In addition, inhibitory peptide administration of pathogen binding to CD28 is now under clinical trial, which is a very promising therapeutic method [15]. However, the prognosis of most patients with NSTI is poor. Up to 20% of NSTI require amputation and 30% of patients have mild to severe organ dysfunctions [16, 17].

Outcomes of NSTI significantly vary depending on initial diagnosis time and therapeutic methods. Misdiagnosis or delayed treatments of NSTI at an early stage frequently occurred because NSTI symptoms sometimes resemble clinical features of other soft-tissue infections and have no specific diagnostic biomarkers [12, 18, 19]. Previous reports have presented some risk factors for the outcome of NSTI, including higher age, underlying diseases, aberrant laboratory findings, and clinical characteristics [20–22]. However, real risk factors of NSTI outcome remain undefined. The present study was conducted to assess the risk factors for mortality and amputation in NSTI.

## 2. Materials and Methods

### 2.1. Patients

We retrospectively reviewed and analyzed the medical records of 111 NSTI patients who were diagnosed and treated in our hospital from 1 January 1999 to 31 December 2018 for this study. Study variables for outcome evaluation included age, gender, underlying diseases, imaging data, microbiological culture of blood or wounds, laboratory findings, organ functions, and therapeutic approaches. The inclusion criteria are as follows: (1) all patients were 18 years or older and (2) all patients were hospitalized in our department. The exclusion criteria are as follows: (1) patients were diagnosed with abscesses and phlegmons and (2) the outpatients were not included in this study. Risk factors evaluation for mortality and amputation was also determined according to LRINEC score [23], which was calculated based on serum C-reaction protein (CRP), WBC, Hb, serum sodium, creatinine, and glucose levels.

### 2.2. Laboratory Findings

The basic laboratory findings for this study included WBC count, Hb, HCT, CRP, serum sodium (Na), urea nitrogen (BUN), creatinine (Cr), blood glucose, blood lactate, procalcitonin (PCT), and international normalized ratio (INR) for prothrombin time test in emergency and hospitalization.

### 2.3. Microorganism Culture in Wound or Blood

Bacterial culture from peripheral blood and wound soft tissues covered *Baumannii, Staphylococcus*, *Klebsiella pneumonia*, and *Escherichia coli* strains growth.

### 2.4. Imaging Examinations

The image studies included echocardiography, computed tomography angiography (CTA), computed tomography (CT), magnetic resonance imaging (MRI), and routine ultrasound in the heart, chest, and arteries and veins of the extremities.

### 2.5. Managements

Managements of NSTI included surgical debridement intervention, empirical broad-spectrum antimicrobial use, and protection of dysfunctional organs. Intravenous immunoglobulin (IVIG), hyperbaric oxygen therapy (HBOT), continuous renal replacement therapy (CRRT) or hemodialysis, and ventilator use were based on disease status following NSTI management protocol.

### 2.6. Outcome

We focused on the chances of mortality and amputation at 90 days after surgery, antibiotic therapy, and protective treatments of organs.

### 2.7. Statistical Analyses

Quantitative data are presented as median (interquartile range, IQR) or mean ± standard deviations (SD). Qualitative data are reported as percentages (%). The prevalence of events presents with frequency and cumulative frequency. Variable comparisons among different groups were performed using the Student's *t*-test or the Mann–Whitney test. For risk factors evaluation of mortality and amputation in NSTI, we first screened using the univariate logistic regression analysis. Then, multivariate logistic regression analysis was used combined with *P* value and 95% CI for the calculation of variables at optimal AUC cut-off value with high sensitivity and specificity to determine the final risk factors. *P* < 0.05 was considered as a significant difference.

## 3. Results

### 3.1. Clinical Features

To identify risk factors for mortality and amputation in NSTI, we first selected eligible patients, who were 18 years or older and hospitalized in our department. As shown in Table 1, the clinical characteristics of NSTI included age, gender, surgery, ICU frequency, the underlying diseases, LRINEC score, mortality rate, and amputation rate. The average age (mean ± standard deviation (SD), range) was 59.50 ± 14.26 years (i.e., 22 to 87 years), and the proportion of patients with >60 years was 54.95%. Male and female patients were eighty-three cases (74.77%) and twenty-eight cases (25.23%), respectively. Among these patients, twenty-two cases (22/111, 19.82%) were hospitalized to ICU at admission. Patients with hypertension, diabetes mellitus, hyperlipidemia, kidney dysfunctions, systemic lupus erythematosus (SLE), chronic obstructive pulmonary disease (COPD), and cancer were 14 (12.61%), 67 (60.36%), 26 (23.42%), 15 (13.51%), 3 (2.7%), 5 (4.5%), 2 (1.8%), and 7 (6.31%), respectively. Most patients had only one lesion (96, 86.49%), which occurred in the feet (49, 44.14%), the scrotum/perineum (22, 19.82%), the lower extremities (24, 21.62%), and other body parts (7, 6.31%). Fifteen patients had multiple lesions (15, 13.5%). The mean LRINEC score (mean ± SD) was 4.20 ± 3.05 (range 0–12). Ten patients (28.57%) had received at least one surgical debridement. Eleven dead cases (11/111, 9.91%) and 38 amputation cases (38/111, 34.23%) were recorded at 90 days after admission. These data indicated that patients with NSTI had frequently underlying diseases, at least one wound when they were initially admitted.

### 3.2. Laboratory and Imaging Findings

Peripheral blood counts and biochemistry data at admission are shown in Table 2. These biomarkers were used to compare later therapeutic effects. We also documented imaging data at admission (Table 3), including echocardiography, computed tomography angiography (CTA) in the extremities, regular or enhanced CT, MRI, chest or extremities X-ray, and ultrasound. The results showed that there were positive findings in less than 50% of patients.

### 3.3. Microorganism Culture in Wound or Blood

NSTIs are frequently involved in various microorganism infections. Therefore, we routinely performed microorganism cultures in wounds or blood. As shown in Table 4, positive rates in *Baumannii*, *Staphylococcus*, *Klebsiella pneumonia*, and *Escherichia coli* were 10.91% (12 cases), 16.36% (18 cases), 11.82% (13 cases), and 8.18% (9 cases), respectively. Infectious chances with more than one kind of bacteria were 70%, and the rate of noninfection was 30%. These results revealed that an antibiotic regimen may be necessary because most patients have at least one bacterial infection.

### 3.4. Managements

**Figure 1:**
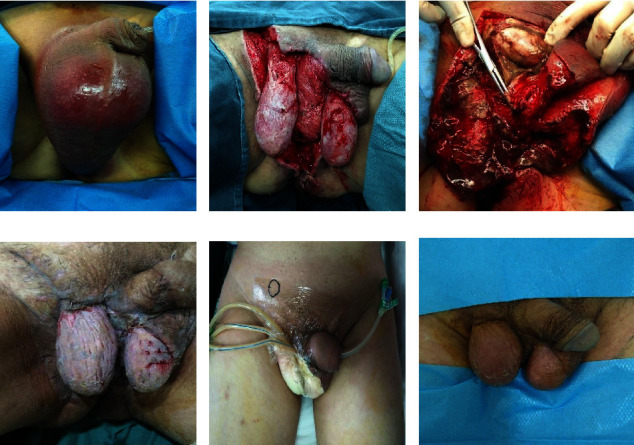
Surgical debridement procedure in a patient with NSTI. (a–f) All steps from initial surgery to end of surgery.

**Figure 2:**
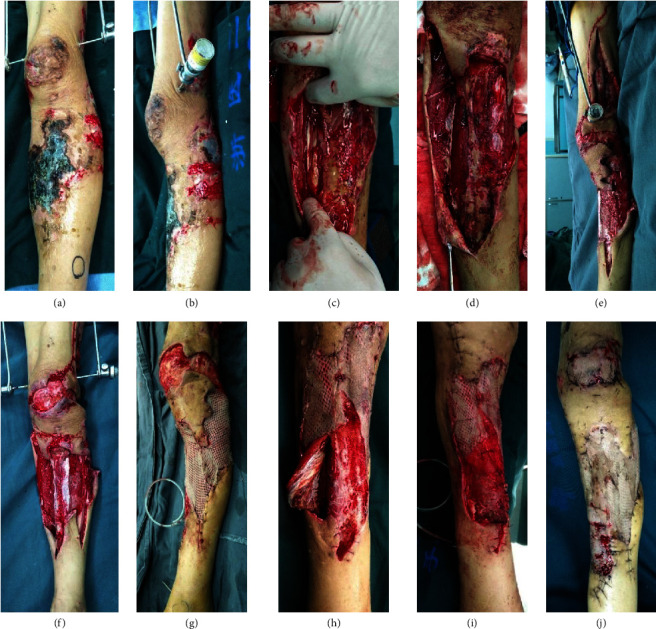
Surgical management of a patient with necrosis of skin and soft tissue in the lower extremity after avulsion. (a, b) Necrosis of skin and soft tissue in the left lower extremity. (c, d) Necrosis of tendons and muscles during debridement. (e, f) Fresh wound and local exposure tibia. (g) After 3 negative pressure therapy. (h, i) Skin grafting over tibia. (j) Recovery of the wound.

After patients were admitted to the hospital, we performed radical surgical debridement intervention and antibiotics administration within 24 hours. Surgical procedures are shown in Figures 1 and 2. The first patient was diagnosed with Fournier gangrene (FG) because he had extensive necrosis of soft tissues in the scrotum (Figure 1(a)). We performed radical surgical debridement (Figures 1(b) and 1(c)), and the damaged area gradually recovered (Figures 1(d) and 1(f)). The second patient was a 56-year-old man with diabetes mellitus. He had necrosis of skin and soft tissue in the lower extremities (Figures 2(a) and 2(b)) because of a car accident. We also found necrosis of tendons and muscles during debridement (Figures 2(c) and 2(d)), fresh wounds (Figures 2(e) and 2(f)), and local exposure tibia (Figure 2(g)). After 3 negative pressure therapies, he experienced skin grafting over the tibia (Figures 2(g) and 2(h)) and was finally recovered (Figure 2(j)). The detailed therapeutic methods and times from emergency or hospitalized clinics are shown in Table 5. A total of 111 patients have experienced surgical debridement in hospitalization. Some patients had more than one surgery, ventilator, or norepinephrine use or lived in the ICU.

### 3.5. Risk Factor Analysis for NSTI Mortality

**Figure 3:**
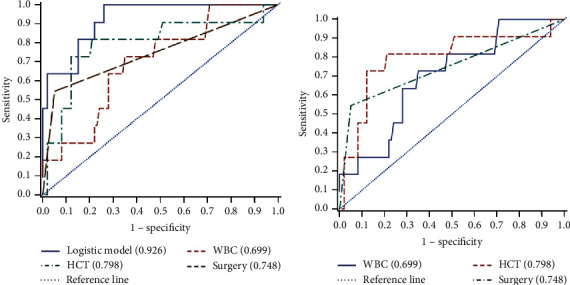
AUC of WBC, HCT, and surgery at admission in nonsurvivor patients with NSTI. (a) ROC plots show results of WBC, HCT, and surgery for predicting mortality in the logistic model. (b) ROC plots show results of WBC, HCT, and surgery for predicting mortality without the logistic model. NSTI: necrotizing soft-tissue infections; ROC: receiver operating characteristics; AUC: area under the ROC curve; WBC: white blood cell; HCT: hematocrit.

The mortality rate of NSTI patients is high. We performed univariate and multivariable logistic analyses for risk factors. LRINEC score was a key factor (Table 6). In this study, 11 of 111 patients died at 90 days after admission. LRINEC score of live patients was significantly lower than that of dead patients (*P* < 0.05). Laboratory data such as CRP, WBC, lactate, and PCT of live patients were dramatically lower than that of the dead patients (*P* < 0.05). In contrast, Hb and HCT of live patients were higher than that of the dead patients (Table 7). We also performed ROC analysis of patients with mortality rate (Table 8; Figures 3(a) and 3(b)), showing the area under the curve (AUC) for WBC, HCT, and surgery with or without the logistic model. The results showed that the AUC, 95% CI, and p value of each variable were 0.699, 0.54–0.95, *P* = 0.0117 for WBC; 0.798, 0.63–0.97, and *P* = 0.0006 for HCT; 0.745, 0.59–0.90, and *P* = 0.0018 for surgery, respectively (Table 9). These results indicated that high WBC, low HCT value, and surgery at admission were critical factors for mortality prediction.

### 3.6. Risk Factors of Amputation for NSTI Patient

**Figure 4:**
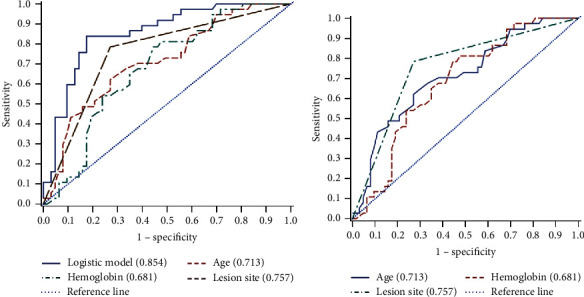
ROC from patient's age, hemoglobin, and wounds for predicting amputation. (a) ROC plots show results of age, hemoglobin, and wounds for predicting amputation in the logistic model. (b) ROC plots show results of age, hemoglobin, and wounds for predicting amputation without the logistic model. NSTI: necrotizing soft-tissue infections; ROC: receiver operating characteristics.

Amputation is a severe outcome of NSTI. To assess risk factors for amputation, we analyzed different data in clinical factors and laboratory findings. We found that higher age, low Hb, and multiple wound sites are positively correlated with amputation (Figure 4). The AUC value can predict amputation using age, hemoglobin, and wound sites as variables with the logistic model (Figure 4(a)) or without the logistic model (Figure 4(b)). The AUCs of age, hemoglobin, and wound sites were 0.713, 0.681, and 0.757 in the logistic model, respectively (Table 10). We also performed a ROC comparison and the Mann–Whitney test. *P* values and 95% CI of age, hemoglobin, and wound sites for amputation prediction were 0.11–0.32 and *P* < 0.0001; 0.08–0.29 and *P*=0.0007; and 0.17–0.34 and *P* < 0.0001, respectively (Table 11). These results revealed that old age, low hemoglobin, and multiple wound sites at admission were critical markers for amputation prediction.

## 4. Discussion

NSTIs are severe and life-threatening diseases, which are frequently misdiagnosed for other diseases because of no typical symptoms [5, 16, 19]. Therefore, the identification of risk factors for outcomes of NSTI is a critical issue. Here, we performed a single center cohort study of 111 patients and reported that 11 of 111 NSTI (9.9%) patients were dead and 38 of 111 patients (34.2%) were amputated at 90 days after admission. LRINEC score, WBC, HCT, and surgery were key indicators for mortality prediction. Higher age, hemoglobin, and multiple wound sites were critical factors for predicting amputation.

The studies showed that the overall mortality rate of NSTI was up to 35% [24, 25]. The reasons for the high mortality rate vary from one hospital to another [20, 26, 27] but one factor may be severe sepsis [28]. The mortality rate in this cohort study was 9.9% (11/111), which was lower than that of the other reports [29–31] but close to other results [7, 8, 32, 33]. Our low mortality may be because of early surgical intervention, active antibiotics use, IVTG, and HBOT administration. We identified that the LRINEC score was an important marker. This result is consistent with other studies [32, 34, 35]. Hua et al. and the other team also reported that patients aged >75 years old had a high mortality rate [3, 7, 8]. The other team found that patients with underlying diseases such as diabetes, cardiovascular disease, and higher lactate levels were associated with a high mortality rate [7, 36, 37]. Our univariate logistic analysis also showed that lactate in dead patients was significantly higher than that in survival patients (Table 7; *P* < 0.05). High WBC, low HCT, and multiple surgeries may reflect that patients have experienced extensive severe sepsis. Indeed, Madsen et al. [7] reported that higher lactate levels may be associated with septic shock. Therefore, if patients with NSTI have these abnormal parameters, then physicians must pay more attention to these patients.

Other interesting findings in this study revealed that higher age, low hemoglobin, and multiple wounds were strongly associated with a high amputation rate. Previous reports indicated that higher age was relevant to high mortality, especially for patients who had underlying diseases [3, 7, 28]. These apparent biomarkers showed that the patients had low immunity because of old age. Low hemoglobin and multiple wounds indicated that NSTI had severe infections. Therefore, early radical surgical debridement and antibiotic use are key factors for protecting amputation. Other reports also indicated that early surgery and antibiotic therapy were very important for amputation [1, 38, 39]. The study also revealed that inhibitory peptide administration of pathogen binding to CD28 can protect organ functions in NSTI [15]. Here, our study showed that *Baumannii*, *Staphylococcus*, *Klebsiella pneumonia*, and *Escherichia coli* strains had a high positive infectious rate. The study indicated that group *A. streptococcus* and *Staphylococcus aureus* infections were especially associated with the lower extremities [7, 31]. These results confirmed that early antibiotic therapy and surgery are critical methods for avoiding amputation and death [31].

This study had few limitations which are as follows: (I) current results were from a single hospital with a retrospective study. This may cause a selection bias and limit the evaluation of relationships between risk factors and outcomes. (II) The total patients were 111 cases in this study. Small sample sizes may affect multivariate regression analysis. Sample size limitation was also reported in other studies because NSTI is a rare disease [1, 2, 40]. To confirm the present findings, we plan to expand our study to multiple hospitals with more participants. (III) NSTI is a severe disease. Its treatment protocols are hard and identical because of complex clinical symptoms and various laboratory findings. This limitation may affect outcomes of NSTI. (IV) The present study followed up for 90 days. We counted dead and amputated patients at this cut-off time. This follow-up time may affect study conclusions.

## 5. Conclusion

The present study revealed that high LRINEC scores, elevated WBC, low HCT, and multiple surgeries were associated with mortality. Higher age, low hemoglobin, and multiple wounds are key markers for indicating amputation. Early antibiotic therapy and surgery are important procedures for avoiding amputation and mortality.

## Figures and Tables

**Table 1:** Demographic characteristics of the patients with NSTI.

Variables		Patients (*n*)	Frequency (%)	Cumulative frequency (%)
Age (year old)	>60	61	54.95	54.95
	≤60	50	45.05	100

Gender	M	83	74.77	74.77
	F	28	23.23	100

ICU admission	Y	22	19.82	19.82
	N	89	80.18	100

Underlying diseases	HTN	14	12.61	12.61
	DM	67	60.36	60.36
	Hyperlipidemia	26	23.42	2.42
	Kidney dysfunction	15	13.51	13.51
	SLE	3	2.70	2.70
	COPD	2	1.80	1.80
	Cancer	7	6.31	6.31

Wound number and locations	1	96	86.49	86.49
	≥2	15	13.51	100
	Multiple	9	8.11	8.11
	Others	7	6.31	14.41
	Lower extremities	24	21.62	36.04
	Scrotum/perineum	22	19.82	55.86
	Feet	49	44.14	100

LRINEC score		111	0–12 (range)	4.2 ± 3.04 (mean ± SD)

Emergency surgery	Y	10	28.57	28.57
	N	25	71.43	100

Mortality rate	Y	11	9.91	9.91
	N	100	90.09	100

Amputation rate	Y	38	34.23	34.23
	N	73	65.77	100

*n*, number; NSTIs, necrotizing soft-tissue infections; Y, yes; N, no; ICU, intensive care unit; HTN, hypertension; DM, diabetes mellitus; SLE, systemic lupus erythematosus; COPD, chronic obstructive pulmonary disease; LRINEC, laboratory risk indicator for necrotizing fasciitis.

**Table 2:** Laboratory findings of blood in 111 NSTI patients.

Variable	*N*	Mean	SD	Minimum	Maximum	Range	25^th^percentile	Median	75^th^percentile	Missing
CRP	111	111.88	111.33	1	515	514	20	65.5	197	0
WBC	111	11.15	8.49	0.3	80	79.7	7	9	13.3	0
Hb	111	94.42	35.16	5.4	162	156.6	76	97	117	0
HCT	111	308.53	70.27	152	479	327	266	309	354	0
Sodium	111	135.94	10.22	42.8	147.4	104.6	133.8	137.8	140.7	0
BUN	111	8.61	7.59	1.27	42.57	41.3	4.45	6.07	9.53	0
Cr	111	95.97	114.83	0.33	733	732.67	50	63	83	0
Glucose	111	9.21	10.43	1.62	95	93.38	5.12	7.29	9.31	0
Lactate	77	1.92	1.87	0.5	15.95	15.45	1.2	1.5	2.1	34
PCT	111	3.2	8.28	0.01	59.89	59.88	0.07	0.38	1.99	0
INR	109	1.24	0.91	0.88	10.5	9.62	1.04	1.12	1.21	2

NSTIs, necrotizing soft-tissue infections; CRP, C-response protein; WBC, white blood cell; Hb, hemoglobin; HCT, hematocrit; BUN, blood urea nitrogen; Cr, creatinine; PCT, procalcitonin; INR, international normalized ratio for prothrombin time test.

**Table 3:** Image studies in 111 NSTI patients.

Examination	Results	Frequency (%)	Cumulative frequency (%)
Echocardiography test	N	63 (56.76)	63 (56.76)
	Y	48 (43.24)	111 (100.0)

Bi/tricuspid valve regurgitation	Y	13 (27.08)	13 (27.08)
	N	35 (72.92)	48 (100.0)

Extremities CTA test	N	67 (72.04)	67 (72.04)
	Y	26 (27.96)	93 (100.0)

Extremities vascular ultrasound	N	49 (52.69)	49 (52.69)
	Y	44 (47.31)	93 (100.0)

Extremities vascular stent	Y	5 (5.38)	5 (5.38)
	N	88 (94.62)	93 (100.0)

Emergency CT	Y	20 (18.02)	20 (18.02)
	N	91 (81.98)	111 (100.0)

Emergency MRI	Y	8 (7.21)	8 (7.21)
	N	103 (92.79)	111 (100.0)

Extremities regular X-ray	Y	16 (17.20)	16 (17.20)
	N	77 (82.80)	93 (100.0)

Chest CT or X-ray	Y	75 (67.57)	75 (67.57)
	N	36 (32.43)	111 (100.0)

Extremities CT or enhanced CT	Y	20 (21.51)	20 (21.51)
	N	73 (78.49)	93 (100.0)

Extremities MRI or enhanced MRI	Y	20 (18.02)	20 (18.02)
	N	91 (81.98)	111 (100.0)

Y, yes; N, no; CTA, computed tomography angiography; CT, computed tomography; MRI, magnetic resonance imaging.

**Table 4:** Bacterial culture features of NSTI patients.

Bacteria culture	Results	Frequency (%)	Cumulative frequency (%)
Bacteria culture	N	1 (0.90)	1 (0.90)
	Y	110 (99.10)	111 (100.0)

Bacteria amount	0	33 (30.00)	33 (30.00)
	1	41 (37.27)	74 (67.27)
	2	22 (20.00)	96 (87.27)
	3	10 (9.09)	106 (96.36)
	4	4 (3.64)	110 (100.0)

Bacteria count	0	33 (31.13)	33 (31.13)
	1	41 (38.68)	74 (69.81)
	≥2	32 (30.19)	106 (100.0)

*Baumannii*	Positive	12 (10.91)	12 (10.91)
	Negative	98 (89.09)	110 (100.0)

*Staphylococcus*	Positive	18 (16.36)	18 (16.36)
	Negative	92 (83.64)	110 (100.0)

*Klebsiella pneumonia*	Positive	13 (11.82)	13 (11.82)
	Negative	97 (88.18)	110 (100.0)

*Escherichia coli*	Positive	9 (8.18)	9 (8.18)
	Negative	101 (91.82)	110 (100.0)

**Table 5:** Management of the patients with NSTI.

Variable	*N*	Mean ± SD	Minimum	Maximum	Range	Median
Emergency to surgery time (d)	10	8.4 ± 3.98	4	15	11	6.75
Numbers of surgery	111	2.23 ± 1.82	0	8	8	2
Initial time (d)	94	51.2 ± 65.34	2	365	363	28
1^st^ to 2^nd^ time (d)	58	7.74 ± 4.98	3	30	27	7
Ventilator use (d)	21	6 ± 6.75	1	30	29	4
Norepinephrine use (d)	14	2.86 ± 2.11	1	9	8	1
ICU time (d)	22	17.32 ± 22.51	1	85	84	4

d, days; NSTIs, necrotizing soft-tissue infections.

**Table 6:** LRINEC score comparison of live and dead patients.

Variable	Group	Size (missing)	Mean ± SD	Median (interquartile range)	Range (minimum and maximum)	Test method	*χ* ^2^(*T* or *Z*)	*P*value
LRINEC	Live	100 (0)	4.02 ± 3.00	3.00 (2.00, 6.00)	12 (0, 12)	Wilcoxon two-sample test	1.971	0.049
	Dead	11 (0)	5.82 ± 3.03	6.00 (3.00, 9.00)	8 (2, 10)			
	Difference (row 1-row 2)		−1.8	95% CI (−3.69, 0.09)				

LRINEC, laboratory risk indicator for necrotizing fasciitis; SD, standard deviation.

**Table 7:** Laboratory findings of live and dead patients with NSTI.

Variable	Group	Size (missing)	Mean ± SD	Median (interquartile)	Range (minimum and maximum)	*χ* ^2^	Statistics (*T* or *Z*)	*P*value
CRP	Live	100 (0)	105.98 ± 112.11	60.50 (16.00, 174.00)	514 (1, 515)	Wilcoxon two-sample test	2.3	0.021
	Dead	11 (0)	165.54 ± 91.56	145.40 (89.10, 240.00)	286.3 (33.7, 320)			
	Differences (row 1-row 2)		−59.56	95% CI (−129.06, 9.94)				

WBC	Live	100 (0)	10.26 ± 5.24	8.70 (6.55, 12.80)	25.3 (0.3, 25.6)	Wilcoxon two-sample test	2.152	0.031
	Dead	11 (0)	19.24 ± 21.01	11.80 (9.00, 18.60)	72.9 (7.1, 80)			
	Range (row 1-row 2)		−8.98	95% CI (−23.11, 5.16)				

Hb	Live	100 (0)	96.11 ± 36.07	101.00 (87.00, 118.50)	156.6 (5.4, 162)	Wilcoxon two-sample test	−2.601	0.009
	Dead	11 (0)	79.09 ± 20.87	76.00 (69.00, 86.00)	77 (55, 132)			
	Differences (row 1-row 2)		17.02	95% CI (1.75, 32.29)				

HCT•	Live	100 (0)	315.64 ± 66.56	310.00 (279.00, 360.00)	327 (152, 479)	Wilcoxon two-sample test	−3.227	0.001
	Dead	11 (0)	243.91 ± 73.21	228.00 (188.00, 267.00)	263 (165, 428)			
	Differences (row 1-row 2)		71.73	95% CI (29.42, 114.04)				

Sodium	Live	100 (0)	136.07 ± 10.55	137.90 (134.00, 140.50)	104.6 (42.8, 147.4)	Wilcoxon two-sample test	−1.145	0.252
	Dead	11 (0)	134.82 ± 6.81	134.00 (130.00, 140.80)	22 (125, 147)			
	Differences (row 1-row 2)		1.25	95% CI (−5.21, 7.71)				

BUN	Live	100 (0)	7.92 ± 6.29	5.99 (4.33, 9.32)	32.46 (1.27, 33.73)	Wilcoxon two-sample test	1.934	0.053
	Dead	11 (0)	14.85 ± 13.93	7.30 (6.30, 24.24)	39.69 (2.88, 42.57)			
	Differences (row 1-row 2)		−6.93	95% CI (−16.34, 2.47)				

Cr	Live	100 (0)	95.97 ± 119.56	63.00 (48.00, 80.00)	732.67 (0.33, 733)	Wilcoxon two-sample test	1.209	0.227
	Dead	11 (0)	96.00 ± 59.40	82.00 (59.00, 112.00)	175 (34, 209)			
	Differences (row 1-row 2)		−0.03	95% CI (−44.88, 44.82)				

Glucose	Live	100 (0)	9.22 ± 10.80	7.17 (5.06, 9.61)	93.38 (1.62, 95)	Wilcoxon two-sample test	0.271	0.786
	Dead	11 (0)	9.09 ± 6.41	7.61 (5.43, 8.66)	23.3 (2.46, 25.76)			
	Differences (row 1-row 2)		0.13	95% CI (−4.54, 4.80)				

Lactate	Live	66 (34)	1.89 ± 2.00	1.45 (1.20, 2.10)	15.45 (0.5, 15.95)	Wilcoxon two-sample test	2.005	0.045
	Dead	11 (0)	2.06 ± 0.68	1.90 (1.60, 2.90)	1.9 (1.1, 3)			
	Differences (row 1-row 2)		−0.17	95% CI (−0.82, 0.47)				

PCT	Live	100 (0)	3.08 ± 8.51	0.35 (0.05, 1.60)	59.88 (0.01, 59.89)	Wilcoxon two-sample test	2.192	0.028
	Dead	11 (0)	4.29 ± 5.96	1.81 (0.32, 6.87)	18.05 (0.18, 18.23)			

NSTIs, necrotizing soft-tissue infections; SD, standard derivation; CRP, C-response protein; WBC, white blood cell; Hb, hemoglobin; HCT, hematocrit; BUN, blood urea nitrogen; Cr, creatinine; PCT, procalcitonin.

**Table 8:** ROC model of NSTI patient mortality.

ROC curve	Mann−Whitney				Somers' D (Gini)	Gamma	Tau-a	Cut-off	Sensitivity	Specificity
	AUC	SD	95% CI							
Model	0.9255	0.0335	0.8599	0.9910	0.8509	0.8509	0.1533	0.042	1	0.73
WBC	0.6986	0.0788	0.5442	0.8531	0.3973	0.3991	0.0716	11.7	0.63	0.72
HCT	0.7977	0.0869	0.6275	0.9680	0.5955	0.5971	0.1073	250	0.88	0.72
Surgery	0.7477	0.0795	0.5919	0.9035	0.4955	0.9160	0.0893	—	0.545	0.950
ROC4	0.5000	0	0.5000	0.5000	0		0			

NSTIs, necrotizing soft-tissue infections; ROC, receiver operating characteristics; AUC, area under the ROC curve; SD, standard derivation; CI, confidence interval; WBC, white blood cell; HCT, hematocrit.

**Table 9:** Comparison of ROC in different parameters of dead patients.

ROC comparison and Mann−Whitney test						
Comparison	Estimate	SD	95% CI		*χ* ^2^	*P* value
Model-0.5	0.4255	0.0335	0.3599	0.4910	161.6852	<0.0001
WBC-0.5	0.1986	0.0788	0.0442	0.3531	6.3513	0.0117
HCT-0.5	0.2977	0.0869	0.1275	0.4680	11.7498	0.0006
Surgery-0.5	0.2477	0.0795	0.0919	0.4035	9.7129	0.0018

NSTIs, necrotizing soft-tissue infections; ROC, receiver operating characteristics; SD, standard derivation; CI, confidence interval; WBC, white blood cell; HCT, hematocrit.

**Table 10:** ROC and AUC of patients with NTSI for amputation.

ROC curve	Mann–Whitney				Somers' D (Gini)	Gamma	Tau-a	Cut-off	Sensitivity	Specificity
	AUC	SD	95% CI							
Model	0.8535	0.0390	0.7771	0.9299	0.7070	0.7073	0.3329	0.044	0.838	0.809
Age	0.7126	0.0533	0.6082	0.8170	0.4251	0.4326	0.2002	63.5	0.622	0.73
Hb	0.6810	0.0535	0.5761	0.7859	0.3621	0.3651	0.1705	105	0.556	0.784
Wounds	0.7570	0.0444	0.6699	0.8440	0.5139	0.8150	0.2420	—	0.784	0.730
ROC4	0.5000	0	0.5000	0.5000	0		0			

NSTIs, necrotizing soft-tissue infections; ROC, receiver operating characteristics; AUC, area under the ROC curve; SD, standard derivation; CI, confidence interval; Hb, hemoglobin.

**Table 11:** ROC comparison of amputation NSTI.

ROC comparison and Mann–Whitney test						
Comparison	Estimate	SD	95% CI		*χ* ^2^	*P* value
Model-0.5	0.3535	0.0390	0.2771	0.4299	82.3149	<0.0001
Age-0.5	0.2126	0.0533	0.1082	0.3170	15.9265	<0.0001
Hb-0.5	0.1810	0.0535	0.0761	0.2859	11.4390	0.0007
Wounds-0.5	0.2570	0.0444	0.1699	0.3440	33.4975	<0.0001

NSTIs, necrotizing soft-tissue infections; ROC, receiver operating characteristics; SD, standard derivation; CI, confidence interval; Hb, hemoglobin.

## Data Availability

The data used to support the findings of the study are available from the corresponding author upon request.
